# Understanding secondary school students’ intentions to learn artificial intelligence: a multigroup structural equation modeling analysis

**DOI:** 10.3389/fpsyg.2026.1833613

**Published:** 2026-06-23

**Authors:** Yuyu Gu, Bingfu Xiong, Ruxue Li, Zhehao Pan, Yipin Huang, Yingying Pan

**Affiliations:** 1 Department of Education, Korea University, Seoul, Republic of Korea; 2 College of Education, Wenzhou University, Chashan University Town, Wenzhou, Zhejiang, China; 3 Wenzhou Polytechnic, Chashan University Town, Wenzhou, Zhejiang, China

**Keywords:** AI learning, intention to learn, K-12 education, multigroup analysis, theory of planned behavior

## Abstract

As artificial intelligence (AI) continues to play an expanding role in everyday activities, AI-related knowledge and skills are recognized as essential 21st-century competencies. Although K-12 AI curricula have been prioritized by educational authorities worldwide, limited research has examined the motivational mechanisms that underpin students’ intentions to engage in AI learning. The present study broadens the Theory of Planned Behavior by integrating self-efficacy, attitude toward using AI, subjective norms, perceived usefulness, and AI literacy to examine students’ intention to participate in AI learning at the secondary level. The study also examines whether gender, grade level, school location, and extracurricular AI learning experience shape these relationships. Participants consisted of 632 secondary school students from Zhejiang Province, China. Students’ intention to learn AI was significantly explained by self-efficacy, attitudes toward AI use, and perceived usefulness according to SEM results. Self-efficacy and perceived usefulness also exerted significant positive effects on attitudes toward AI use. By contrast, while AI literacy and subjective norms significantly affected self-efficacy and perceived usefulness, their direct effects on attitudes toward AI use were not significant. The multigroup analysis further revealed that gender did not function as a significant moderator, whereas grade level, school location, and extracurricular AI learning experience did. Practical implications for educators and policymakers emerge from these findings, particularly for the development of inclusive AI learning programs.

## 1 Introduction

Artificial intelligence (AI) technologies are increasingly embedded in everyday life and are profoundly reshaping global labor markets. Estimates from the McKinsey Global Institute suggest that in about 60% of occupations, at least 30% of constituent work activities could be automated by 2030 (Manyika et al., 2017). Against this backdrop, the abilities to understand, critically engage with, and apply AI have become essential 21st-century competencies, enabling individuals to sustain competitiveness and adaptability in rapidly changing social and economic contexts (Ng et al., 2021; Southworth et al., 2023). In response to these developments, international organizations and national governments have increasingly advocated the integration of AI education into K–12 curricula. For example, the UNESCO report *K-12 AI Curricula: A Mapping of Government-Endorsed AI Curricula* (UNESCO, 2022) reviewed international policy and curricular practices and called on governments to incorporate AI education into national K–12 strategic agendas. Several countries, including the United Kingdom, Canada, Portugal, South Korea, and Finland, have already implemented relevant initiatives (Liu et al., 2025). In China, the Ministry of Education issued a notice in 2024 on strengthening AI education in primary and secondary schools, with the goal of achieving near-universal implementation of AI education in these schools by 2030 (Ministry of Education of the People’s Republic of China, 2024).

AI education involves developing learners’ understanding of fundamental AI concepts, their ability to use AI tools effectively, and their capacity to critically evaluate the ethical implications of AI for individuals and society (Ng et al., 2021; Southworth et al., 2023). For secondary school students, AI learning is particularly important, as this developmental stage is marked by rapid growth in abstract reasoning, digital literacy, and ethical responsibility (Semerci Şahin et al., 2025; Özbay, 2026). Their AI learning experiences during this period can profoundly shape students’ future technological awareness, career choices, and social participation. Nevertheless, the abstract nature of AI concepts and the technical demands associated with AI-related learning activities may pose substantial challenges for young learners (Zhang H. et al., 2025). Therefore, students’ learning intentions and motivation become important factors for effective engagement with AI learning. Prior studies have largely concentrated on instructional theories, curriculum design, technological tools, and the effectiveness of learning content (e.g., Yue et al., 2022; Li et al., 2024; Wu et al., 2024), with relatively few explaining the behavioral mechanisms underlying students’ intentions to learn AI from the perspective of learners’ internal motivation. When learning tasks are complex and abstract, even well-designed curricula may have limited effectiveness if learners lack intrinsic motivation and willingness to learn (Chai et al., 2020). Therefore, clarifying the motivational mechanisms that shape secondary school students’ intentions to learn AI constitutes a critical issue in advancing AI education research and addresses an important gap in the existing literature.

The Theory of Planned Behavior (TPB) provides a widely used theoretical lens for behavioral intentions. The predictive validity of attitudes, subjective norms, and perceived behavioral control for behavior has been validated across various technology-enhanced learning contexts (Sing et al., 2022; Liu and Wang, 2024; Al-Qaysi et al., 2025). Unlike general academic learning, AI learning requires students not only to understand complex concepts but also to navigate the uncertainty associated with rapid technological development. Accordingly, students’ intentions to learn AI may depend not only on their attitudes toward AI learning but also on their underlying cognitive beliefs. In this regard, perceived usefulness, which refers to students’ judgments regarding the value of AI learning, and self-efficacy, a key dimension of perceived behavioral control reflecting students’ self-assessment of their abilities, jointly shape students’ behavioral intentions regarding AI learning (Chai et al., 2020, 2021; Chen et al., 2020). Moreover, AI literacy refers to the foundational knowledge and abilities required to understand, apply, and evaluate artificial intelligence, as well as to recognize and reflect on AI-related ethical issues (Ng et al., 2021). Prior studies have shown that AI literacy influences students’ attitudes toward AI learning, self-efficacy, and perceived usefulness (Chai et al., 2020; Chiu et al., 2024), suggesting that it constitutes an important learner-level factor in AI learning environments. Accordingly, this study adapted the TPB framework to the AI learning context by integrating self-efficacy (SE), attitude toward using AI (ATU), subjective norms (SN), perceived usefulness (PU), and AI literacy (LIT) to examine secondary school students’ behavioral intentions (BI) to learn AI.

In addition, Fishbein and Ajzen (2011) identified the moderating role of background factors in shaping BI, noting that individuals with similar attitudes, perceived norms, and perceptions of control may still behave differently. In the context of secondary school AI learning, factors including gender, grade level, school location, and extracurricular experience capture systematic variations in students’ cognitive development, access to educational resources, and prior knowledge accumulation. Such differences may fundamentally shape the mechanisms and strength through which motivational variables influence learning intentions. Hu et al. (2022) provided empirical evidence showing the moderating roles of student gender and age differences in adolescents’ intentions to learn game-based programming, underscoring the need to account for individual background differences in technology learning settings.

Grounded in the TPB framework and informed by the distinctive characteristics of AI learning, this study adopts a learner-centered motivational perspective to examine the relationships among SE, ATU, SN, PU, LIT, and BI. It further investigates the moderating roles of key background variables, including gender, grade level, school location, and extracurricular AI learning experience, in shaping students’ intentions to learn AI. This study aims to clarify the motivational mechanisms underlying secondary school students’ AI learning intentions and to identify potential variations in these pathways across different student groups. The findings are expected to provide empirical evidence for designing targeted pedagogical interventions and support strategies in AI education.

## 2 Literature review and hypotheses development

### 2.1 Theory of planned behavior

Building on the Theory of Reasoned Action (TRA), the Theory of Planned Behavior (TPB) was developed to account for behaviors influenced by factors beyond complete volitional control (Ajzen, 1991). Within this framework, Ajzen (1991) conceptualized behavior as being determined by BI, which is jointly shaped by three key constructs: attitudes, subjective norms, and perceived behavioral control. The TPB framework posits that favorable attitudes, perceived social norms, and perceived behavioral control collectively promote individuals’ engagement in a specific behavior. The TPB offers a parsimonious yet comprehensive framework with strong explanatory and predictive power for human behavior, demonstrating robust explanatory and predictive validity (Wang et al., 2025). Its utility has been confirmed across diverse contexts, including entrepreneurial intention, environmental protection (Wang et al., 2019), and technology adoption (Azizi and Khatony, 2019). Given its contextual adaptability and structural flexibility, researchers have continually refined and extended the TPB to address educational phenomena. Building on this theoretical foundation and considering the distinctive characteristics of AI education, the present study extends the TPB to develop and validate a model that explains secondary school students’ intentions to learn AI.

### 2.2 Core constructs of the TPB

In TPB, BI is strongly influenced by ATU, which reflects individuals’ evaluative orientation toward a given behavior (Ajzen, 1985). In the present study, attitude reflects students’ evaluative perceptions of AI use, indicating whether they hold favorable views of learning and applying AI. Grounded in the TPB, students’ positive attitudes toward the use of AI may enhance their perceptions of AI learning as meaningful, valuable, and engaging, which in turn strengthens their intentions to learn AI. Numerous studies in educational contexts have confirmed the predictive effect of ATU on students’ BI (Arkorful et al., 2020; Chai et al., 2020; Wu et al., 2022). Hence, the hypothesis was developed as follows:

*H*1: ATU positively influences BI.

PBC reflects an individual’s judgment of the controllability of a particular behavior based on their prior experiences and anticipated obstacles. Although Ajzen (2002) noted that behavioral control consists of both SE and controllability, empirical studies have frequently operationalized PBC primarily through measures of SE or confidence (Fishbein and Ajzen, 2011; Chai et al., 2020). SE is defined as the belief in one’s capability to carry out actions needed to accomplish objectives (Bandura, 1997). In this study, SE is defined as students’ confidence in understanding AI concepts and their belief that they can succeed if they devote sufficient effort. SE affects learners’ formation of ATU, capacity to acquire skills, selection of activities, and BI (Liaw and Huang, 2013; Chai et al., 2020, 2021). Based on this reasoning, hypotheses were developed as follows:

*H*2: SE positively influences BI.

*H*3: SE positively influences ATU.

SN refers to individuals’ perceptions of social pressure from important others (e.g., parents, teachers, and peers) regarding whether to perform a behavior, thereby influencing behavioral intention (Ajzen, 1991). Nevertheless, in the emerging context of AI education, the complexity of AI technologies and the abstract nature of AI concepts present considerable cognitive difficulties for secondary school students (Micheuz, 2020). Given students’ limited familiarity with AI, social expectations may exert a more direct influence on their cognitive appraisals of AI learning than on their behavioral intentions. Empirical evidence further supports this theoretical refinement. Chai et al. (2020) showed a significant effect of SN on PU, attitudes, and SE, while no direct effect was observed on students’ AI learning intentions. Likewise, Wang et al. (2025) showed that while SN significantly affected students’ attitudes towards and PBC of their use of generative AI technologies, it exerted no direct influence on BI (Saxena and Doleck, 2023). Accordingly, this study argues that, in the context of AI education, the effect of SN on students’ behavioral intentions may operate primarily through learners’ cognitive variables rather than through the direct pathway proposed in the conventional TPB. Based on this reasoning, hypotheses were developed as follows:

*H*4: SN positively influences SE.

*H*5: SN positively influences ATU.

*H*6: SN positively influences PU.

### 2.3 Extended factors of the TPB

The Technology Acceptance Model (TAM) posits that PU defined as the degree to which using a system enhances one’s performance, and perceived ease of use, or the degree to which a system is easy to understand and use, serve as primary drivers of users’ attitudes, which in turn lead to behavioral intention (Davis, 1989). Nevertheless, empirical studies have shown that perceived ease of use often does not directly predict users’ attitudes toward or intentions to use technology (Chen et al., 2020; Wang et al., 2020; Hu et al., 2022). Researchers have pointed out that even when technology is perceived as complex, users will still adopt it due to its PU; this is particularly true for digital natives (Al-Adwan et al., 2023). Given that secondary school students are digital natives with relatively high receptivity and adaptability to emerging technologies, this study focused exclusively on the effect of PU on learning intention. The positive influence of PU on attitudes and adoption intentions in educational technology contexts has been well established (Chen et al., 2020; Wang et al., 2020; Alfadda and Mahdi, 2021; Hu et al., 2022; Al-Adwan et al., 2023). Based on this reasoning, hypotheses were developed as follows:

*H*7: PU positively influences BI.

*H*8: PU positively influences ATU.

As AI increasingly emerges as a core technology driving societal transformation, LIT has become a fundamental competency required in the 21st century. In their exploratory review, Ng et al. (2021) conceptualized LIT as a multidimensional construct encompassing four core capabilities: understanding AI, using and applying AI, evaluating and creating AI, and demonstrating awareness of AI ethics. As students’ LIT increases, they are better able to recognize the practical value of AI and apply it effectively in problem-solving contexts (Chai et al., 2021; Wang et al., 2023). With increased AI-related knowledge and skills, they become more confident and positive about learning AI technologies. Higher levels of LIT enable students to better understand the usefulness of AI and to solve problems more effectively (Chai et al., 2021; Wang et al., 2023). Previous studies have shown that LIT positively influences students’ perceptions of the usefulness of AI technologies (Chai et al., 2020; Al-Abdullatif and Alsubaie, 2024) and serves as an important predictor of SE and attitudes (Chai et al., 2020, 2021; Dai et al., 2020; Wang et al., 2025). Accordingly, hypotheses were developed as follows:

*H*9: LIT positively influences SE.

*H*10: LIT positively influences ATU.

*H*11: LIT positively influences PU.

### 2.4 Moderating variables: gender, grade level, school location, and extracurricular experience in learning AI

The moderating role of gender in education has long been discussed, particularly in relation to technology. Reviews by Whitley (1997) and Cai et al. (2017) noted that previous studies generally indicated that males are more likely than females to express positive attitudes toward technology use, thereby confirming and reinforcing the view that gender-based differences are evident across various technology-related domains. Ameen et al. (2019) found that the relationship between PU and students’ BI to use e-learning systems varies by gender. However, Hu et al. (2022), in examining adolescents’ intention to engage in game-based programming, reported that the effects of PU on BI and SE on BI were not moderated by gender. The findings of Su and Chen (2022) also revealed that gender had no significant moderating effect on Chinese university students’ use of learning management systems. Such inconsistencies in moderating effects highlight the need for further research on how gender influences AI learning intentions among secondary school students.

At the same time, researchers have argued that higher-grade students use information and communication technologies (ICT) more frequently than lower-grade students, both inside and outside school, and therefore possess greater experience with technology (Gu et al., 2013). Lin (2011) investigated learning experience plays a moderating role in shaping continued e-learning intention. A stronger association between attitudes toward e-learning and continued learning intention was observed among users with extensive learning experience. By contrast, Strzelecki (2024) reported that grade level had no significant effect on AI adoption among higher education students in the context of ChatGPT use. These divergent findings underscore the complexity of how grade level and prior learning experience influence technology use intention.

Moreover, substantial urban–rural inequalities in access to educational resources continue to characterize the Chinese context, with urban students being more likely than their rural counterparts to have frequent exposure to AI and other advanced technologies (Zhang C. et al., 2025). Wang and Yin (2025) further reported that the association between anthropomorphism and challenge appraisal in GenAI adoption varied by family location, with rural students exhibiting a more pronounced negative relationship. This may be because rural communities often place greater emphasis on established social norms and community-oriented practices rather than technological progress, and limited awareness of AI tends to lead to more pronounced negative evaluations. This indicates that regional differences not only affect students’ exposure to AI but also shape their beliefs about it. Therefore, we proposed gender, grade level, school location, and extracurricular experience of AI learning as moderating variables in the paths.

**Figure 1:**
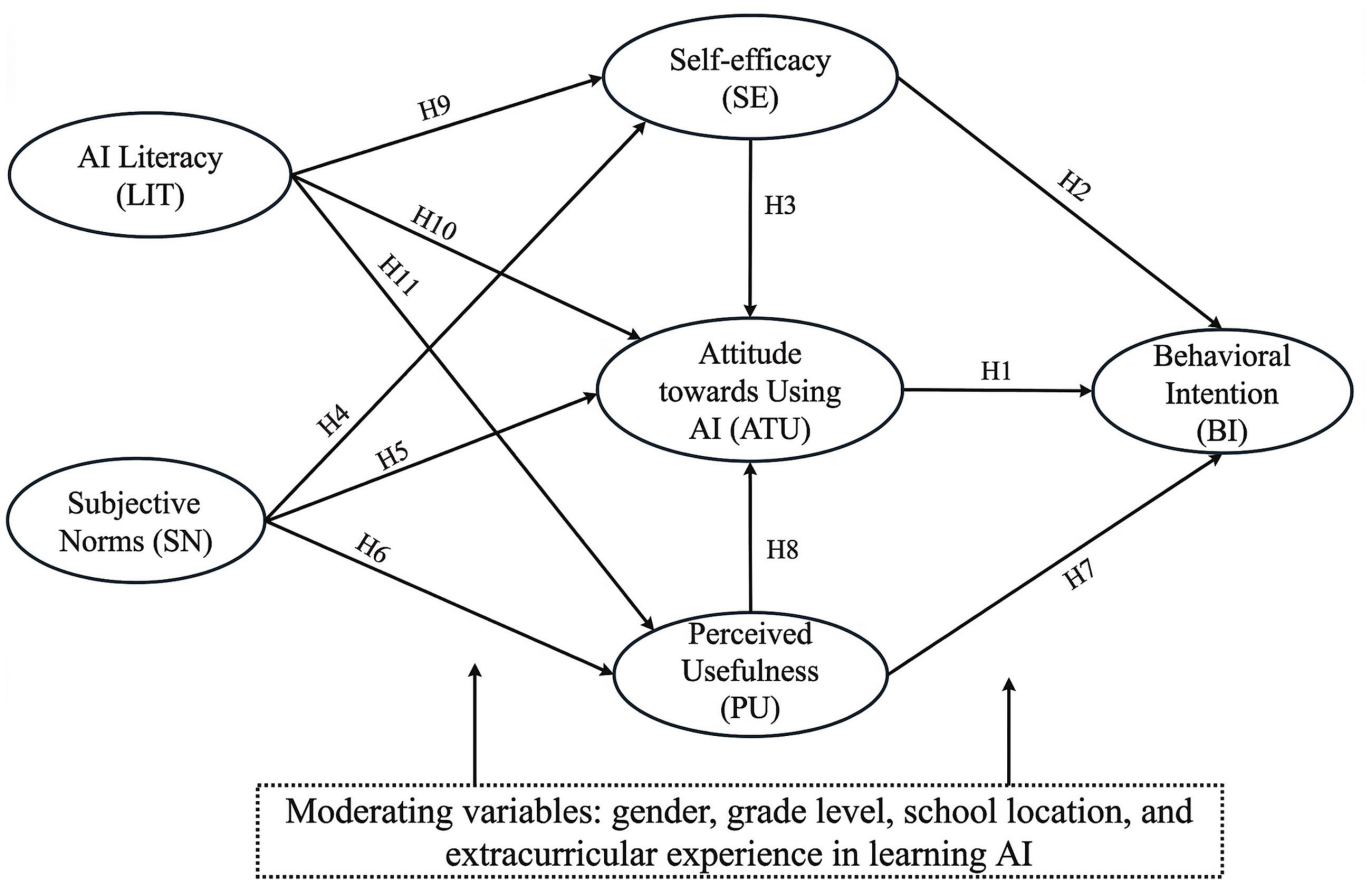
Extended TPB-based model.

Overall, this study extends and validates a TPB model within AI education settings to explore the motivational mechanisms of AI learning intentions among students at the secondary level. As illustrated in Figure 1, the model incorporates attitude towards using AI, SE, SN, LIT, and PU as predictors to examine their relationships with learning intention, with gender, school location, grade level, and extracurricular experience in AI learning moderating all connections between constructs.

## 3 Methods

### 3.1 Participants

Participants were drawn from Zhejiang Province, China, using a combination of convenience and snowball sampling methods. The survey was administered through Wenjuanxing, an online questionnaire platform commonly used in China, after obtaining informed consent from students and their parents or guardians. In total, 632 valid questionnaires were collected, and the demographic profile is summarized in Table 1. Among the respondents, 318 students (50.32%) were male and 314 (49.68%) were female. In terms of grade distribution, 182 students (28.80%) were in grade 7, 367 (58.07%) were in grade 8, and 83 (13.13%) were in grade 9. Regarding school location, 304 students (48.10%) attended urban schools, while 328 (51.90%) were from rural schools. In addition, 163 students (25.79%) reported having extracurricular experience in learning AI, whereas 469 students (74.21%) had no such experience.

**Table 1:** Demographic information of participants.

Characteristic	Classification	Number	Proportion (%)
Gender	Male	318	50.32
	Female	314	49.68
Grade level	Grade 7	182	28.80
	Grade 8	367	58.07
	Grade 9	83	13.13
School location	Urban	304	48.10
	Rural	328	51.90
Extracurricular experience of learning AI	Yes	163	25.79
	No	469	74.21

### 3.2 Measures

AI learning intentions among students at the secondary level were assessed using a questionnaire composed of two parts. Demographic characteristics, comprising gender, grade level, school location, and extracurricular experience of AI learning, were assessed in the first section. The questionnaire’s second section assessed six constructs using 31 items adapted from prior research, all rated on a 5-point Likert scale (1 = strongly disagree, 5 = strongly agree). LIT was measured using an instrument adapted from Wang et al. (2023). The instrument comprised four dimensions, Awareness, Usage, Evaluation, and Ethics, with three items per dimension, yielding a total of 12 items. The questionnaires measuring SN (4 items), PU (4 items), and ATU (3 items) were adapted from the scales used in Chai et al. (2020). SE (4 items) and intention to learn AI (4 items) were measured using the scales from Chai et al. (2021).

To ensure linguistic and conceptual equivalence between the original English scales and their Chinese versions, this study adopted a translation and back-translation procedure. Two professional translators familiar with the purpose and content of the scales independently translated the original English items into Chinese. Subsequently, two independent translators who were blind to the original English versions back-translated the Chinese items into English. The four translators then jointly compared the original English versions, the Chinese translations, and the English back-translations item by item to evaluate consistency in conceptual meaning, semantic accuracy, and technical terminology. Following the translation and back-translation process, two psychology experts and the research team reviewed all Chinese items to assess content appropriateness, linguistic clarity, and comprehensibility for secondary school students. Based on their feedback, several items were further refined. The revised Chinese version was then pilot-tested with a small sample of students to evaluate fluency, clarity, and ease of understanding. Minor adjustments were made based on the feedback, resulting in the final Chinese version of the scales used for formal data collection.

### 3.3 Data analysis

A three-stage data analysis framework was employed in the present study. First, descriptive statistics were computed using SPSS 25, and distributional normality was evaluated via skewness and kurtosis, with values falling within |3| and |8| regarded as indicative of acceptable distributions (Kline, 2016).

In the second step, confirmatory factor analysis (CFA) and structural equation modeling (SEM) were applied to assess the research model, and parameters were estimated using maximum likelihood estimation. Evaluation procedures involved first validating the measurement model with respect to reliability and convergent validity, and discriminant validity followed by structural model testing to examine the relationships among the latent variables. Following the guidelines of Hair et al. (2019), model fit is evaluated by reporting the chi-square statistic and degrees of freedom, together with at least one incremental and one absolute fit index. In this study, the fit indices examined included the chi-square ratio (CMIN/DF), Tucker-Lewis Index (TLI), Comparative Fit Index (CFI), Root Mean Square Error of Approximation (RMSEA), and Standardized Root Mean Square Residual (SRMR), which together offer complementary and non-redundant evidence for assessing model fit. In general, a CMIN/DF value less than 3 is considered indicative of an acceptable model fit (Kline, 2016). Values of TLI and CFI exceeding 0.90 are indicative of satisfactory model fit (Hair et al., 2019; Kline, 2016). In addition, a satisfactory fit is assumed when RMSEA and SRMR values met the recommended thresholds of 0.08 and 0.05, respectively (Kline, 2016).

In the third step, to examine differences in AI learning intention among learners with different backgrounds, multigroup analyses were conducted based on gender (male vs. female), grade level (seventh, eighth, and ninth grades), school location (urban vs. rural), and extracurricular experience in AI learning (yes vs. no). To ensure instrument validity and reliability across groups, measurement invariance was examined before multigroup analyses, based on the four levels outlined by Byrne (2010): configural, metric, scalar, and strict. Changes in model fit were evaluated using ΔCFI, with values ≤ 0.01 suggesting no meaningful deterioration (Cheung and Rensvold, 2002). Measurement invariance having been confirmed, group differences in the structural models were examined through multigroup analysis. Both the second and third steps were carried out using the AMOS 24 software package.

## 4 Results

### 4.1 Descriptive statistics

Descriptive statistics in Table 2 indicate that acceptable skewness (−0.315 to 0.219) and kurtosis (−0.735 to 0.657) values were observed for all constructs, indicating that the data reasonably satisfied the assumption of normality.

**Table 2:** Descriptive statistics of all constructs.

Variables	Overall (*N* = 632)			
	Mean	SD	Skewness	Kurtosis
SE	3.629	0.822	0.219	−0.347
PU	3.864	0.796	−0.007	−0.683
ATU	3.776	0.816	0.150	−0.735
BI	3.649	0.821	0.184	−0.494
SN	3.616	0.830	0.152	−0.216
LIT	3.823	0.747	−0.315	0.657

### 4.2 Measurement model testing

Table 3 presents a series of statistical measures, including factor loadings, Cronbach’s alpha, composite reliability (CR), and average variance extracted (AVE). Measurement adequacy was evaluated using Hair et al.’s (2019) guidelines, which recommend factor loadings above 0.50, AVE greater than 0.50, CR values exceeding 0.70, and Cronbach’s alpha values between 0.70 and 0.95. Therefore, the questionnaire used in this study demonstrated acceptable reliability and convergent validity. As a final step in model evaluation, discriminant validity was assessed by examining whether the square roots of AVE values exceeded the inter-construct correlations (Fornell and Larcker, 1981). Table 4 presents the results of the discriminant validity analysis, indicating that all scales in this study satisfied the required criteria.

**Table 3:** Measurement model reliability and convergent validity.

Construct	Number of items	Factor loadings	AVE	CR	Cronbach’s α
SE	4	0.838–0.927	0.796	0.940	0.938
PU	4	0.860–0.923	0.800	0.941	0.942
ATU	3	0.882–0.917	0.807	0.926	0.927
BI	4	0.857–0.886	0.764	0.928	0.928
SN	4	0.788–0.860	0.679	0.894	0.892
LIT	12	0.620–0.857	0.600	0.947	0.946

**Table 4:** Measurement model discriminant validity.

Construct	SE	PU	ATU	BI	SN	LIT
SE	0.892					
PU	0.775^***^	0.894				
ATU	0.871^***^	0.858^***^	0.898			
BI	0.857^***^	0.742^***^	0.820^***^	0.874		
SN	0.814^***^	0.794^***^	0.793^***^	0.761^***^	0.824	
LIT	0.757^***^	0.748^***^	0.734^***^	0.743^***^	0.764^***^	0.775

Diagonal elements (in italics) are the square roots of the AVE; off-diagonal elements are the correlation estimates; ^***^p < 0.001.

Finally, a satisfactory overall model fit for the measurement model was supported by the CFA results, with fit indices of CMIN/DF = 4.488, TLI = 0.917, CFI = 0.925, RMSEA = 0.074, and SRMR = 0.031. Despite the CMIN/DF value marginally exceeding 3, its value below 5 was deemed acceptable given the sensitivity of the χ^2^ statistic to large sample sizes (Hayduk, 1987).

### 4.3 Results of the structural model

**Figure 2:**
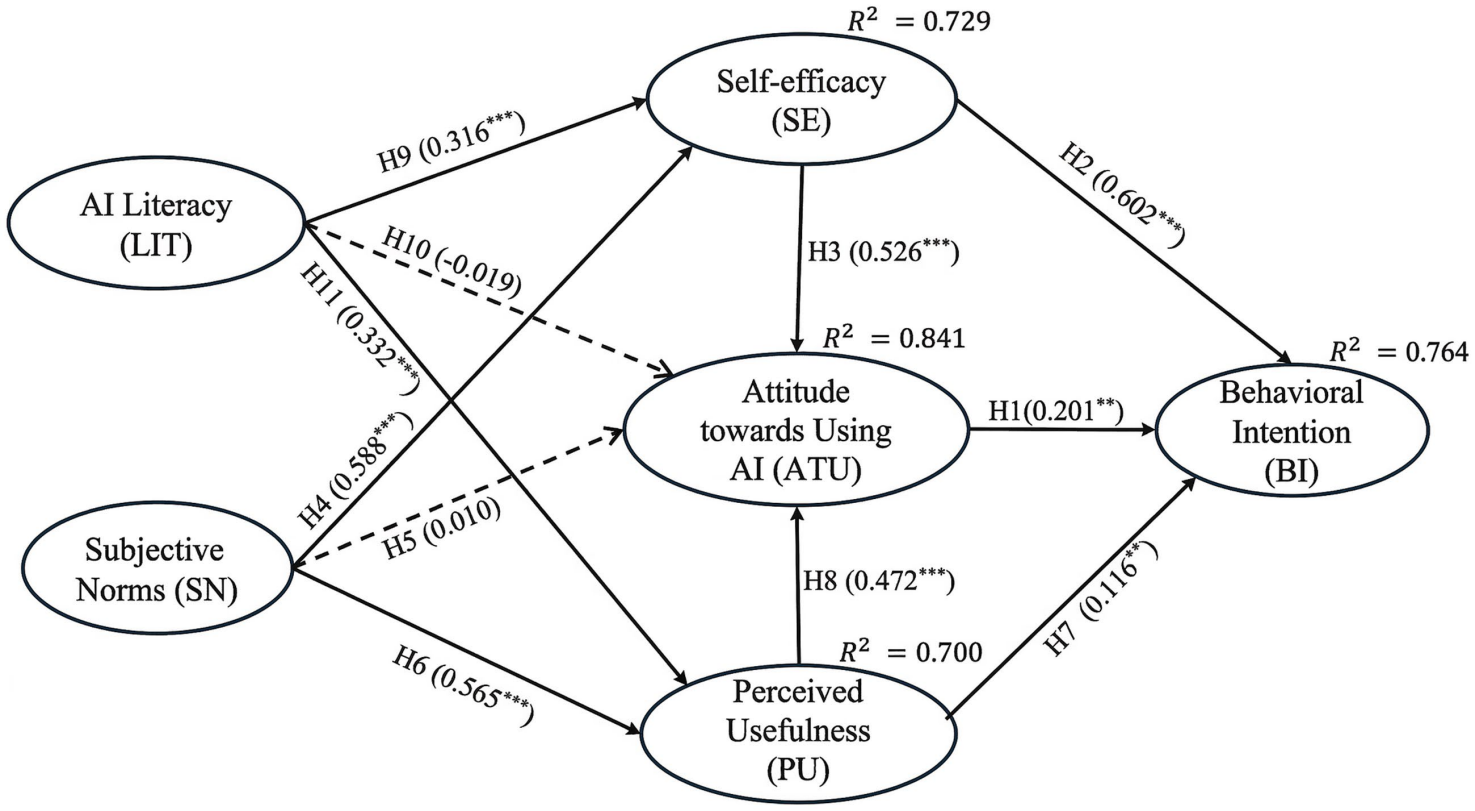
Results of the research model.

The model demonstrated acceptable fit, as indicated by the following indices: CMIN/DF = 4.557, TLI = 0.915, CFI = 0.923, RMSEA = 0.075, and SRMR = 0.033. The path analysis results in Table 5 indicate that nine of the 11 hypotheses received support. Figure 2 displays the standardized path coefficients, distinguishing significant relationships with solid lines and nonsignificant relationships with dashed lines. SE (*β* = 0.602, *p* < 0.001), ATU (*β* = 0.201, *p* < 0.01), and PU (*β* = 0.116, *p* < 0.01) all had significant positive effects on BI. The model accounted for 76.4% of the variance in BI, supporting H1, H2, and H7. LIT (*β* = 0.316, *p* < 0.001) and SN (*β* = 0.588, *p* < 0.001) had significant positive effects on SE. The model explained 72.9% of the variance in SE, providing support for H4 and H9. LIT (*β* = 0.321, *p* < 0.001) and SN (*β* = 0.565, *p* < 0.001) had significant positive effects on PU. Support for H6 and H11 was evidenced by the model’s explanation of 70% of the variance in PU. In addition, SE (*β* = 0.526, *p* < 0.001) and PU (*β* = 0.472, *p* < 0.001) had significant positive effects on ATU, with the model accounting for 84.1% of the variance in ATU. However, LIT (*β* = −0.019, *p* > 0.05) and SN (*β* = 0.010, *p* > 0.05) did not have significant effects on ATU. Accordingly, H3 and H8 were supported, whereas H5 and H10 were rejected.

**Table 5:** Hypothesis testing results.

Hypothesis	Path	Standardized estimate	SE	*t*-value	Result
H1	ATU→BI	0.201	0.073	2.683^**^	Accepted
H2	SE→BI	0.602	0.054	10.673^***^	Accepted
H3	SE→ATU	0.526	0.047	10.873^***^	Accepted
H4	SN→SE	0.588	0.054	12.119^***^	Accepted
H5	SN→ATU	0.010	0.063	0.167	Rejected
H6	SN→PU	0.565	0.053	11.491^***^	Accepted
H7	PU→BI	0.116	0.050	2.297^**^	Accepted
H8	PU→ATU	0.472	0.045	10.572^***^	Accepted
H9	LIT→SE	0.316	0.053	7.000^***^	Accepted
H10	LIT→ATU	−0.019	0.046	−0.468	Rejected
H11	LIT→PU	0.321	0.053	6.889^***^	Accepted

^***^p < 0.001; ^**^p < 0.01; ^*^p < 0.05.

### 4.4 Multigroup analysis of moderating effects

#### 4.4.1 Multigroup analysis with gender as a moderating variable

The comparability of construct interpretation across gender groups was established through measurement invariance testing prior to multigroup analysis. The model comparison results summarized in Table 6 show that all ΔCFI values were ≤ 0.01, thus supporting configural, metric, scalar, and strict invariances. Table 7 summarizes the results of the multigroup analysis. While some differences in specific structural paths were noted across gender groups, they did not attain statistical significance (*p* > 0.05). Therefore, gender-based moderation of the model’s structural relationships was not supported.

**Table 6:** Measurement invariance testing results.

Model	χ^2^/*df*	CFI	RMSEA	ΔCFI	Result
M1: Configural Invariance	3.174	0.907	0.059		
M2: Metric Invariance	3.102	0.908	0.058	0.001	Accepted
M3: Scalar Invariance	3.112	0.904	0.058	0.004	Accepted
M4: Strict Invariance	3.085	0.900	0.058	0.004	Accepted

**Table 7:** Multigroup analysis across gender.

Path	Standardized path coefficient			*p*
	Male	Female	Δχ^2^	
	(*n* = 318)	(*n* = 314)	Δ*df* = 1	
LIT→SE	0.361^***^	0.255^***^	0.084	0.772
LIT→ATU	−0.020	−0.034	0.022	0.882
LIT→PU	0.382^***^	0.254^***^	0.123	0.726
SN→SE	0.549^***^	0.641^***^	1.954	0.162
SN→ATU	−0.133^*^	0.278^**^	2.474	0.116
SN→PU	0.514^***^	0.614^***^	0.616	0.433
PU→ATU	0.469^***^	0.400^***^	0.438	0.508
PU→BI	0.073	0.146^*^	0.047	0.828
SE→ATU	0.682^***^	0.326^***^	0.595	0.440
SE→BI	0.606^***^	0.582^***^	0.121	0.728
ATU→BI	0.233	0.197^*^	0.168	0.682

^***^p < 0.001; ^**^p < 0.01; ^*^p < 0.05.

#### 4.4.2 Multigroup analysis with grade level as a moderating variable

As summarized in Table 8, metric and scalar invariance were supported, whereas strict invariance was not achieved (ΔCFI > 0.01). As noted by Vandenberg and Lance (2000), achieving strict invariance is often challenging, and meaningful cross-group comparisons remain valid when only a few items violate the invariance assumption. Following Hair et al. (2019), partial invariance was adopted as an acceptable alternative to strict invariance. Accordingly, the strict constraints of several items, including three from LIT, one from ATU, and two from SN, were released to establish a partial strict invariance model (M5). A comparison of M5 and the scalar invariance model (M3) revealed no statistically significant difference (ΔCFI ≤ 0.01; Table 8).

**Table 8:** Measurement invariance testing results.

Model	χ^2^/*df*	CFI	RMSEA	ΔCFI	Result
M1: Configural Invariance	3.203	0.867	0.059		
M2: Metric Invariance	3.110	0.867	0.058	0.000	Accepted
M3: Scalar Invariance	3.070	0.864	0.057	0.003	Accepted
M4: Strict Invariance	3.149	0.849	0.058	0.015	Rejected
M5: Partial strict invariance	3.087	0.854	0.058	0.010	Accepted

Table 9 presents the multigroup analysis results, showing that grade level significantly moderated three structural paths: from LIT to SE, PU to BI, and ATU to BI. Specifically, the effect of LIT on SE was significantly positive among Grade 7 (*β* = 0.518, *p* < 0.001) and Grade 8 students (*β* = 0.148, *p* < 0.01), but not significant in Grade 9 (*β* = 0.213, *p* > 0.05). The path from PU to BI showed a significantly positive effect in Grade 8 (*β* = 0.214, *p* < 0.01) and Grade 9 (*β* = 0.308, *p* < 0.001), whereas it was negative and nonsignificant in Grade 7 (*β* = −0.109, *p* > 0.05). The effect of ATU on BI was significantly positive in Grade 7 (*β* = 0.488, *p* < 0.01) and Grade 9 (*β* = 0.282, *p* < 0.01), but negative and nonsignificant in Grade 8 (*β* = −0.032, *p* > 0.05; Table 9).

**Table 9:** Multigroup analysis across grade level.

Path	Standardized path coefficient				*p*
	Grade 7	Grade 8	Grade 9	Δχ^2^	
	(*n* = 182)	(*n* = 367)	(*n* = 83)	Δ*df* = 1	
LIT→SE	0.518^***^	0.148^**^	0.213	5.549	0.018
LIT→ATU	−0.159	−0.048	0.344^**^	0.109	0.741
LIT→PU	0.454^***^	0.174^*^	0.301^*^	1.234	0.267
SN→SE	0.392^***^	0.739^***^	0.701^***^	2.034	0.154
SN→ATU	−0.011	0.104	−0.092	2.638	0.104
SN→PU	0.420^***^	0.690^***^	0.652^***^	0.750	0.386
PU→ATU	0.522^***^	0.434^***^	0.341^*^	3.072	0.080
PU→BI	−0.109	0.214^**^	0.308^***^	12.022	0.001
SE→ATU	0.633^***^	0.491^***^	0.404^***^	0.811	0.368
SE→BI	0.443^***^	0.738^***^	0.443^***^	1.976	0.160
ATU→BI	0.488^**^	−0.032	0.282^**^	5.779	0.016

^***^p < 0.001; ^**^p < 0.01; ^*^p < 0.05.

#### 4.4.3 Multigroup analysis with school location as a moderating variable

Table 10 summarizes the measurement invariance results with school location as the moderating variable. All ΔCFI values were ≤ 0.01, indicating that configural, metric, scalar, and strict invariances were achieved. Table 11 presents the multigroup analysis results, showing that school location moderates the paths from LIT to SE and ATU, as well as the paths from PU and SE to ATU. Specifically, the effect of LIT on SE was significantly positive for both urban (*β* = 0.230, *p* < 0.001) and rural students (*β* = 0.394, *p* < 0.001). Although the effect of LIT on ATU differed slightly between groups, it was nonsignificant in both urban (*β* = −0.047, *p* > 0.05) and rural (*β* = 0.005, *p* > 0.05) samples. The effect of PU on ATU was significantly positive for both the urban (*β* = 0.471, *p* < 0.001) and rural (*β* = 0.525, *p* < 0.001) groups. Likewise, SE exerted a significantly positive influence on ATU in both the urban (*β* = 0.446, *p* < 0.001) and rural (*β* = 0.565, *p* < 0.001) groups.

**Table 10:** Measurement invariance testing results.

Model	χ^2^/*df*	CFI	RMSEA	ΔCFI	Result
M1: Configural Invariance	3.341	0.901	0.061		
M2: Metric Invariance	3.269	0.901	0.060	0.000	Accepted
M3: Scalar Invariance	3.206	0.900	0.059	0.001	Accepted
M4: Strict Invariance	3.157	0.897	0.059	0.003	Accepted

**Table 11:** Multigroup analysis across school location.

Path	Standardized path coefficient			*p*
	Urban	Rural	Δχ^2^	
	(*n* = 304)	(*n* = 328)	Δ*df* = 1	
LIT→SE	0.230^***^	0.394^***^	5.737	0.017
LIT→ATU	−0.047	0.005	4.917	0.027
LIT→PU	0.364^***^	0.253^***^	0.241	0.623
SN→SE	0.663^***^	0.517^***^	0.315	0.575
SN→ATU	0.103	−0.097	1.899	0.168
SN→PU	0.491^***^	0.659^***^	0.555	0.456
PU→ATU	0.471^***^	0.525^***^	5.155	0.023
PU→BI	0.076	0.154^*^	0.077	0.781
SE→ATU	0.446^***^	0.565^***^	4.920	0.027
SE→BI	0.547^***^	0.667^***^	0.044	0.834
ATU→BI	0.264^*^	0.118	0.019	0.890

^***^p < 0.001; ^*^p < 0.05.

#### 4.4.4 Multigroup analysis with extracurricular experience in learning AI as a moderating variable

Table 12 summarizes the measurement invariance results with extracurricular experience in AI learning as the moderating variable. A partial invariance strategy was adopted by releasing the strict invariances of two items from LIT and one from ATU. The comparison of the partial strict invariance model (M5) and the scalar invariance model (M3) revealed no statistically significant difference (ΔCFI ≤ 0.01).

**Table 12:** Measurement invariance testing results.

Model	χ^2^/*df*	CFI	RMSEA	ΔCFI	Result
M1: Configural Invariance	3.458	0.896	0.062		
M2: Metric Invariance	3.385	0.896	0.062	0.000	Accepted
M3: Scalar Invariance	3.382	0.892	0.061	0.004	Accepted
M4: Strict Invariance	3.529	0.880	0.063	0.012	Rejected
M5: Partial strict invariance	3.470	0.883	0.063	0.009	Accepted

Multigroup analysis results presented in Table 13 demonstrated that extracurricular experience in AI learning moderated the paths from LIT, SN, PU, and SE to ATU. The effect of LIT on ATU was nonsignificant for both students with extracurricular experience (*β* = −0.118, *p* > 0.05) and those without such experience (*β* = 0.010, *p* > 0.05). Similarly, the effect of SN on ATU was nonsignificant in both groups with extracurricular experience (*β* = −0.269, *p* > 0.05) and those without such experience (*β* = 0.048, *p* > 0.05). In contrast, PU exhibited a significantly positive effect on ATU in both the group with extracurricular experience (*β* = 0.606, *p* < 0.001) and the group without extracurricular experience (*β* = 0.469, *p* < 0.001). Likewise, SE showed a significantly positive influence on ATU among students with extracurricular experience (*β* = 0.682, *p* < 0.001) and those without (*β* = 0.494, *p* < 0.001).

**Table 13:** Multigroup analysis across extracurricular experience in learning AI.

Path	Standardized path coefficient			*p*
	Yes	No	Δχ^2^	
	(*n* = 163)	(*n* = 469)	Δ*df* = 1	
LIT→SE	0.266^***^	0.341^***^	0.001	0.975
LIT→ATU	−0.118	0.010	8.172	0.004
LIT→PU	0.269^***^	0.356^***^	0.308	0.579
SN→SE	0.690^***^	0.531^***^	0.363	0.547
SN→ATU	−0.269	0.048	9.124	0.003
SN→PU	0.690^***^	0.498^***^	0.026	0.872
PU→ATU	0.606^***^	0.469^***^	5.775	0.016
PU→BI	0.115	0.113	0.005	0.944
SE→ATU	0.682^***^	0.494^***^	5.158	0.023
SE→BI	0.644^***^	0.562^***^	0.018	0.893
ATU→BI	0.219^**^	0.215	0.028	0.867

^***^p < 0.001; ^**^p < 0.01.

## 5 Discussion

By extending the TPB framework, this study incorporated self-efficacy (SE), attitude toward using AI (ATU), subjective norms (SN), perceived usefulness (PU), and AI literacy (LIT) as predictors to explore the underlying mechanisms shaping secondary school students’ behavioral intentions (BI) to learn AI. Based on the empirical analysis, several major conclusions can be drawn.

In alignment with the core tenets of the TPB and a substantial body of empirical evidence, students’ SE, ATU, and PU exerted significant positive influences on their BI (Chai et al., 2020, 2021; Hu et al., 2022; Wu et al., 2022; Al-Adwan et al., 2023). This suggests that secondary school students’ intentions to learn AI are jointly shaped by competence beliefs, value perceptions, and evaluative attitudes. Specifically, students with stronger SE, PU, and ATU tend to report stronger intentions to learn AI. Among these predictors, SE exerted the strongest predictive effect, indicating that in AI learning contexts characterized by a certain degree of abstraction and technical complexity, students’ beliefs about their ability to successfully learn AI may play a more direct role in shaping learning intentions than positive ATU or PU. As Prior et al. (2016) noted, enhancing students’ self-efficacy is an important instructional goal, since students with higher self-efficacy are more likely to increase their engagement in learning.

LIT positively predicted SE and PU, aligning with earlier findings (Chai et al., 2020; Dai et al., 2020; Sing et al., 2022; Al-Abdullatif and Alsubaie, 2024). LIT essentially represents a structured body of prior knowledge that reduces the uncertainty learners encounter when engaging with AI technology and consequently enhances their self-efficacy beliefs. Moreover, LIT enables learners to develop realistic expectations about the technological functions available; the consistency between expectations and actual capabilities further reinforces PU of the technology (Al-Abdullatif and Alsubaie, 2024). The relationship between LIT and ATU observed in this study differs from findings reported in prior research (e.g., Chai et al., 2020). Specifically, LIT did not significantly predict secondary school students’ ATU in the present study. Although Chai et al. (2020) also examined Chinese secondary school students, their participants possessed prior learning experience and had additionally taken part in researcher-provided after-school AI programs. In contrast, the participants in the present study had not received additional AI training. This discrepancy suggests that successful prior learning experiences may be an important condition for translating AI literacy into positive affective attitudes toward AI learning.

Consistent with previous studies, SN had significant positive effects on both SE and PU (Chai et al., 2020; Sing et al., 2022). Notably, in the present study, the predictive power of SN on both SE and PU substantially surpassed that of LIT. Chinese culture and social institutions are deeply rooted in collectivism and Confucianism, in which the attitudes of parents, teachers, and peers are broadly internalized as critical references for individual behavioral decisions (Chan, 2022). Against the macro-level backdrop of national policies actively advancing AI education, normative support from parents, teachers, and schools sends a consistent message of social expectations. In alignment with the interactionist view of environmental and personal factors in social cognitive theory (Bandura, 1986), such social support derived from significant others functions as a valuable external resource, which is pivotal for bolstering students’ learning self-efficacy. Moreover, the group consensus conveyed by SN—that AI learning is inherently valuable—further reinforces students’ perceptions of the usefulness of AI learning. Nevertheless, the non-significant effect of SN on ATU observed in this study differs from findings reported in prior research (Wang et al., 2025). Secondary school students, situated in a critical period of self-identity formation (Erikson, 1968), demonstrate relatively low motivation to comply with external normative pressures due to their psychological and developmental characteristics. Consequently, external expectations may influence cognitive appraisal but may not directly shape affective evaluation, thus failing to effectively translate into positive learning attitudes. By contrast, SE and PU exert significant positive effects on ATU, consistent with earlier studies (Kumar et al., 2020; Wang et al., 2020) and in line with the core propositions of expectancy-value theory (Eccles et al., 1983). According to this theory, individuals’ expectations for success (SE) and their judgments of task value (PU) jointly form the cognitive foundation underlying attitude formation.

Although a substantial body of research has shown that males generally outperform females in attitudes toward technology use, technological literacy, beliefs, and self-efficacy (Hatlevik et al., 2018), other scholars have argued that females hold positive attitudes toward technology use and that the gender gap is narrowing (Cai et al., 2017). The results of this study regarding the moderating role of gender showed that while differences existed in certain path coefficients, multigroup comparisons did not identify significant moderating effects. This finding is consistent with the observed trend, suggesting that as opportunities for technology engagement become more equalized, traditional gender stereotypes are likely to be dismantled.

Moderating effects of grade level were observed on the paths from LIT to SE, PU to BI, and ATU to BI. Specifically, the effect of LIT on SE was significant in Grade 7 and Grade 8 but became nonsignificant in Grade 9. One possible explanation is that as students progress to higher grades, they encounter greater complexity and challenges in AI learning. Experiences of failure may undermine their SE (Bandura, 1997), reducing the marginal impact of LIT on SE. In terms of the effect of PU on BI, the effect was strongest for ninth graders, followed by eighth graders, and non-significant for seventh graders, showing a gradual increase across grade levels. This indicates that as learning experience accumulates, students’ perceptions of AI usefulness become more concrete and translate into learning intentions more effectively. Regarding the effect of ATU on BI, the effect was strongest for seventh graders, followed by ninth graders, and non-significant for eighth graders. For seventh graders, the relatively simple learning content enables positive attitudes to more directly influence learning intentions. When students enter eighth grade, the increased difficulty of learning tasks imposes higher cognitive load (Paas, 1992), weakening the extent to which attitudes translate into intentions. By ninth grade, after continued learning, students’ attitudes become more stable and internalized, and the effect of this pathway reemerges.

School location was found to moderate the relationships between LIT and SE, LIT and ATU, PU and ATU, as well as SE and ATU. A notable finding is that the effect of LIT on SE was stronger among rural students than among urban students. A possible explanation is that these students often face limited technological resources (Wu et al., 2019), and rural students generally exhibit lower levels of LIT. This situation makes rural students with relatively high AI literacy stand out within their peer groups, and this comparative advantage is more easily translated into significant improvements in efficacy beliefs. Although LIT did not significantly affect ATU in either group, the multigroup comparison revealed a significant difference between urban and rural students, suggesting that the process of translating LIT into attitudes may vary by educational context. Moreover, both PU and SE exerted significant positive effects on ATU in both urban and rural groups, with stronger effects observed among rural students. One possible explanation is that while urban students are more frequently exposed to AI applications and their practical value, they are also more aware of ethical concerns such as privacy, bias, and fairness, which may foster skepticism and reduce students’ tendency to accept AI uncritically (Araujo et al., 2020).

Extracurricular AI learning experience also moderated the relationships among LIT, SN, PU, SE, and ATU. The direct paths from both LIT and SN to ATU were non-significant across both groups, yet significant between-group differences were observed, suggesting that extracurricular experience may primarily influence the internalization mechanism through which LIT and SN translate into ATU. In contrast, the paths from PU and SE to ATU were significantly positive in both groups, with stronger effects for students who had out-of-school learning experiences. This finding is consistent with Stoel and Hye Lee (2003), who found that experienced learners tend to develop more comprehensive cognitions about technology and greater confidence in their own abilities. As a result, their perceived usefulness and efficacy beliefs more readily translate into positive attitudes toward AI use. This result further highlights the facilitative role of prior learning experience in shaping positive attitudes toward AI learning.

## 6 Implications

Several notable theoretical and practical implications emerge from the findings of this study. A key theoretical contribution of this study lies in its extension of the TPB framework to explain secondary school students’ intentions toward AI learning. By extending the TPB framework, this study advances AI education research by elucidating how cognitive, attitudinal, and contextual factors collectively influence students’ intentions to learn AI. The results have important practical implications for the effective delivery of AI education at the secondary education level. Teachers should not only focus on transmitting AI-related knowledge, but also on strengthening students’ confidence in learning AI by designing progressively challenging learning tasks, providing timely and constructive feedback, and offering consistent encouragement. Moreover, curricula should emphasize the real-world relevance and social value of AI, encourage collaborative learning through peer groups, and connect AI concepts with students’ everyday experiences and future career aspirations. Such pedagogical practices can enhance students’ perceived usefulness of AI, nurture positive learning attitudes, and ultimately strengthen their intentions to engage with AI learning. In addition, educational resource developers and curriculum designers can incorporate gamified, level-based learning activities into textbooks and digital platforms to help students gradually build their SE. Presenting authentic cases and real-world examples can also enhance students’ awareness of the practical applications of AI, thereby increasing motivation and fostering more positive attitudes toward AI learning.

The multigroup analysis further deepened understanding of contextual differences in AI learning. For lower-grade students, SE was found to be more strongly influenced by LIT, suggesting that instruction at this stage should focus on developing fundamental knowledge and technical skills. As students progress to higher grades, PU becomes a more effective predictor of learning intention, indicating that later instruction should emphasize value recognition, real-world application, and reflective understanding of AI. Positive attitudes, by contrast, exerted their strongest effect on intention among seventh graders, suggesting that nurturing favorable attitudes toward AI is especially important in the early stage of secondary education. School location emerged as a moderating factor, pointing to the importance of adopting differentiated approaches. In rural schools, where access to technological resources remains limited, teachers should prioritize cultivating students’ SE and PU by integrating AI concepts into familiar, contextually relevant examples. Conversely, in urban schools, educators should guide students toward a more critical and reflective understanding of AI, helping them balance enthusiasm for innovation with ethical awareness.

Finally, the moderating effect of extracurricular learning experience underscores the importance of extending AI education beyond the classroom. Students with such experiences were more likely to translate their SE and PU into positive attitudes toward AI learning. This suggests that schools and educational authorities should promote extracurricular opportunities, such as AI-themed workshops, competitions, lectures, and online courses, to enrich students’ learning experiences and strengthen their motivation. By fostering diverse and continuous exposure to AI, these initiatives can help cultivate sustained interest, deeper understanding, and more meaningful engagement among secondary school students.

## 7 Limitations and future research

The interpretation of the results should take several limitations into account. First, all variables were measured using self-reported scales, which may have introduced social desirability bias and common method bias. Future studies could incorporate additional methods, such as interviews and classroom observations, to further validate the findings and enhance the robustness of the conclusions. Second, the sample was drawn exclusively from a single province in China, and snowball and convenience sampling were used, which may constrain the external validity of the findings. Moreover, given China’s collectivist cultural orientation, the functioning of subjective norms in this study may differ from that observed among students in other cultural contexts. Consequently, the generalization of the findings to other provinces, educational levels, and cultural backgrounds should be approached with caution. Future research should consider employing probability sampling or stratified random sampling and conducting cross-group comparisons across multicultural contexts to examine the generalizability of the model. Third, this study primarily used self-efficacy to operationalize perceived behavioral control (PBC). Although this approach is relatively common in educational research, it may not fully capture the controllability dimension of PBC. Future research could adopt a more comprehensive measurement of PBC by incorporating both self-efficacy and controllability, thereby further enhancing the explanatory power of the model. Fourth, this study employed a cross-sectional design, which cannot capture dynamic changes over time. Future research could adopt a longitudinal design to better examine the causal relationships among variables and their developmental trajectories.

## Data Availability

The raw data supporting the conclusions of this article will be made available by the authors, without undue reservation.
